# Improved Energy Supply Regulation in Chronic Hypoxic Mouse Counteracts Hypoxia-Induced Altered Cardiac Energetics

**DOI:** 10.1371/journal.pone.0009306

**Published:** 2010-02-18

**Authors:** Guillaume Calmettes, Véronique Deschodt-Arsac, Gilles Gouspillou, Sylvain Miraux, Bernard Muller, Jean-Michel Franconi, Eric Thiaudiere, Philippe Diolez

**Affiliations:** 1 Laboratoire de Résonance Magnétique des Systèmes Biologiques, UMR 5536 CNRS Université Bordeaux 2, Bordeaux, France; 2 Laboratoire de Pharmacologie, INSERM U885, Université Bordeaux 2, Bordeaux, France; University of Cincinnati, United States of America

## Abstract

**Background:**

Hypoxic states of the cardiovacular system are undoubtedly associated with the most frequent diseases of modern time. Therefore, understanding hypoxic resistance encountered after physiological adaptation such as chronic hypoxia, is crucial to better deal with hypoxic insult. In this study, we examine the role of energetic modifications induced by chronic hypoxia (CH) in the higher tolerance to oxygen deprivation.

**Methodology/Principal Findings:**

Swiss mice were exposed to a simulated altitude of 5500 m in a barochamber for 21 days. Isolated perfused hearts were used to study the effects of a decreased oxygen concentration in the perfusate on contractile performance (RPP) and phosphocreatine (PCr) concentration (assessed by ^31^P-NMR), and to describe the integrated changes in cardiac energetics regulation by using Modular Control Analysis (MoCA). Oxygen reduction induced a concomitant decrease in RPP (−46%) and in [PCr] (−23%) in Control hearts while CH hearts energetics was unchanged. MoCA demonstrated that this adaptation to hypoxia is the direct consequence of the higher responsiveness (elasticity) of ATP production of CH hearts compared with Controls (−1.88±0.38 vs −0.89±0.41, p<0.01) measured under low oxygen perfusion. This higher elasticity induces an improved response of energy supply to cellular energy demand. The result is the conservation of a healthy control pattern of contraction in CH hearts, whereas Control hearts are severely controlled by energy supply.

**Conclusions/Significance:**

As suggested by the present study, the mechanisms responsible for this increase in elasticity and the consequent improved ability of CH heart metabolism to respond to oxygen deprivation could participate to limit the damages induced by hypoxia.

## Introduction

During chronic hypoxia (CH), heart faces an increased workload as a result of the hypoxia-induced pulmonary hypertension under unfavorable conditions of decreased oxygen availability to oxidative cardiac metabolism. Pulmonary hypertension induces a right ventricular hypertrophy that may distort the left ventricle [1]–[3] and impair its function [1], [2]. Along with global heart remodeling, complex structural, hormonal and biochemical modifications occur, concerning both energy-producing and energy-consuming processes in heart cells (see [4], [5]). Studies on hearts of animals exposed to 3-week hypoxia reported an important reduction in mitochondrial mass (measured by the decrease in citrate synthase activity), associated with the reduction of the activities of respiratory chain complexes [6]. In parallel, a perturbation of calcium handling has been observed [7], leading to the decrease in calcium transients and therefore alteration of contractility [7]. Those modifications are concomitant with a decrease in phosphorylated metabolites concentrations in CH hearts, mainly phosphocreatine (PCr) [8], [9]. Altogether, these results evidence the development of a global heart failure in CH hearts [1], [10], [11].

However, adaptation to chronic hypoxia also represents a well-defined and reproducible mean to improve cardiac tolerance to ischemic and/or hypoxic conditions [12]. Indeed, hearts of chronically hypoxic animals develop smaller myocardial infarction [13] and exhibit better functional recovery [14] following ischemia compared with controls. Moreover, incidence and severity of arrhythmia developed during myocardial oxygen deprivation are strongly decreased in hearts from animals submitted to CH by contrast with controls [15].

Although the detailed mechanism remains poorly understood, improved cardiac bioenergetics appears as a potential candidate in this higher resistance of CH hearts to hypoxic insult (see [12], [16]). Particularly, CH-induced mitochondrial modifications such as enhanced expression of uncoupling protein 2 [17], increase in mitochondrial antioxidant enzymes [18], activation of mitoK_ATP_ channel [19], or regulation of cytochrome oxidase by nitric oxide production (see [12], [16]) have been proposed to participate for maintaining resting potential, limit calcium influx, conserve cellular [ATP], and prevent excessive prolongation of action potential duration under hypoxic conditions (see [16]). Still, the overall effect as well as the relative importance of these various modifications induced by CH on heart energetics are still poorly documented, and their comprehension needs the development of experimental strategies aimed at the study of integrated organ physiology and pathophysiology.

In this context, the use of Top-down or Modular approaches of Metabolic Control Analysis (MCA, [20]) allows to overcome the complexity of intracellular regulations and could help to decipher the integration of molecular modifications developed during pathologies [21]. By combining MCA with non-invasive NMR spectroscopy, we are developing a new approach (MoCA, for Modular Control Analysis [22]) to describe the internal regulation of integrative energy metabolism in the intact beating heart. MoCA describes heart energetics on the basis of a supply-demand system (energy-production and -consumption connected by the energetic intermediates). Assessment of changes in [PCr] by ^31^P-NMR spectroscopy and in heart contractile activity allows the quantification of the integrated kinetic response (the elasticity) of supply and demand modules to [PCr] changes. MoCA may potentially provide the full description and quantification of the integrated regulatory effects of any modulation (even complex) of heart contractile activity [22]. Since recent evidence indicates that defects in communication between ATP-producing and ATP-consuming cellular sites are a major factor contributing to energetic deficiency in heart failure [23], tools such as MoCA, allowing study of the integrated cardiovascular function, are of particular interest [24]. Recently, we applied MoCA to study the effects of a 3-week CH on the energetics of contraction of intact perfused mouse hearts [8]. Despite the severe mitochondrial alteration after CH reported previously in the entire heart [6], MoCA evidenced an increased elasticity of the energy-producing processes (mainly mitochondria) in CH hearts leading to an improved response to an increase in energy demand [8]. The beneficial consequence of this surprising modification of mitochondrial regulation for integrative cardiac energetics is an unexpected decrease in the control exerted by energy-production on contraction which represents a clear positive adaptation of oxidative phosphorylation system to CH [8]. However, while this result strengthens the idea that mitochondrial energetics plays a crucial role in heart protection against chronic hypoxia, the direct relationship with CH-induced cardioprotection remains to be investigated. In the present study, we applied MoCA on isolated hearts of Control and CH hearts perfused under conditions of reduced oxygen concentration to investigate the advantages of the increased elasticity of energy-producing processes developed after CH for cardiac function during hypoxia.

## Results

### Changes in Body Weight, Heart Weight and Mitochondrial Content Induced by Chronic Hypoxia

Table 1 presents the modifications of body and heart weight for both Control and CH mice. As previously measured [8], an increase of the total heart weight to body weight ratio was observed in the mice used for this study at the end of a 3-week experimental period of exposure to hypoxia (table 1). This modification is mainly the consequence of the global heart remodeling in response to chronic hypoxia (i.e. increase in right ventricle mass associated with a decrease in left ventricle mass, table 1), also evidenced and described in vivo by us and others with magnetic resonance imaging [1], [3] or doppler echocardiography [25].

**Table 1:** Effect of chronic hypoxia on mouse morphometrics and heart mitochondrial content.

	Control mice (n = 7)	CH mice (n = 6)
BW (g)	30.7±0.8	27.6±1.4*
HW (mg)	132±9	167±6**
RVW (mg)	26±3	68±5**
LVW (mg)	111±17	101±5*
HW/BW (x1000)	4.3±0.3	6.1±0.3**
CS activity (nmol/min/g)	140.5±13.9	115.3±13.7**

BW: Body weight. HW: Heart weight. RVW: Right ventricle weight. LVW: Left ventricle weight. CS: Citrate Synthase.

*p<0.05 and **p<0.01 between Control and CH mice.

Moreover, as suggested by the decrease in citrate synthase activity (table 1), surrogate of mitochondrial content, CH induced a significant decrease in heart mitochondrial content. Similar alteration of mitochondrial content has already been reported in rat heart after CH [6].

### Modular Control Analysis (MoCA) of Hearts Under Low Oxygen

MoCA rationale states that steady states of metabolic systems are the direct consequence of the kinetic responses (elasticities) of the different modules in the system to changes in metabolic intermediates (see methods and [22] for details). In this study, the elasticity analysis was applied under low oxygen perfusion to Control and CH hearts. The mean values of the relative changes in [PCr] and RPP carried out experimentally for the determinations of the elasticities are presented in table 2.

**Table 2:** Experimental relative changes in [PCr] and in contractile activity under low oxygen, measured in Control and CH hearts after specific modulation of energy supply and demand.

		Control mice	CH mice
Reference Steady State	[PCr] (mM)	10.43±1.54	11.39±2.08
	RPP (mmHg/min)	18497±2255	17480±5429
Increase in balloon pressure	Δ[PCr]/[PCr]_i_	−13.5%±5.8	−8.9%±1.6
	ΔRPP/RPP_i_	+15.5%±11.3	+20.0%±8.7
NaCN/IAA inhibition	Δ[PCr]/[PCr]_i_	−39.7%±12.9	−41.9%±8.9
	ΔRPP/RPP_i_	−63.0%±9.8	−60.4%±7.3

i: initial.

Interestingly, the increase in balloon pressure induced a lower decrease in [PCr] (−8.9% vs −13.5%) for a higher increase in RPP (+20.0% vs +15.5%) in CH hearts when compared with Controls (table 2). On the contrary, inhibition of energy-production by NaCN/IAA injection induced comparable changes in [PCr] (−41.9% vs −39.7%) and in RPP (−60.4% vs −63.0%) for CH and Control hearts (table 2).

The resulting elasticities for energy supply and demand modules, calculated from these relative changes, as well as the corresponding repartition of the control in the cardiac bioenergetics system, are reported in table 3 for both Control and CH groups. Under reduced oxygen availability, elasticity of energy supply was twice higher (in absolute value) in CH hearts compared with Controls (−1.88 vs −0.89, p<0.01, table 3), whereas the elasticity of energy demand was identical for both groups (2.09 vs 1.73, non significantly different, table 3). The direct consequence is that (i) not only the control exerted by energy supply on contraction is much lower in CH hearts than in Control (46 vs 69, p<0.05, table 3), but more importantly, (ii) that the repartition of the control on contraction in CH hearts is almost equally shared between energy supply and demand (46%–54%, table 3) whereas in Control hearts control of contraction is clearly shifted toward energy supply (69%–31%, table 3).

**Table 3:** Elasticities and corresponding control coefficients of energy supply and demand modules in Control and CH hearts under low oxygen conditions.

		Control mice (n = 7)	CH mice (n = 6)
Elasticities	Energy-supply	−0.89±0.41	−1.88±0.38**
	Energy-demand	2.09±0.66	1.73±0.62
Control coefficients (%)	Energy-supply	69±14	46±7*
	Energy-demand	31±14	54±7*

*P<0.05 and **P<0.01 between Control and CH hearts.

### Modular Regulation Analysis of the Effect of Low Oxygen

Combined with previous MoCA analysis of Control and CH hearts under normal conditions of oxygen availability [8], the present results allows the quantification of the internal effects (regulation analysis) of a decrease in oxygen on mouse cardiac bioenergetics for both Control and CH groups. Indeed, considering values from [8] as reference for mouse heart energetics, the measurement of the changes induced by any external effectors (e.g. hormone, pathology, etc) offers the possibility to access to internal regulations induced by this external effector on heart energetics (i.e. effects of decrease in oxygen availability in this study).

**Figure 1:**
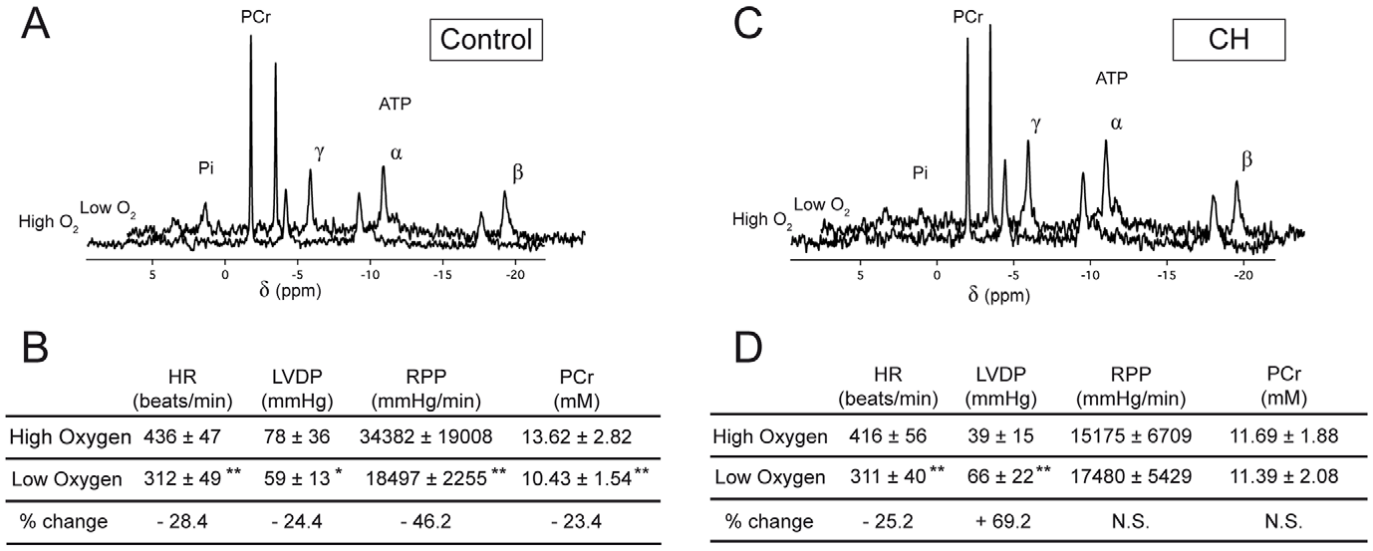
Energetic parameters of the perfused hearts of Control and CH mice under high and low oxygen conditions. Representative ^31^P-NMR spectra, corresponding PCr concentrations, and contraction parameters under high [8] and low oxygen perfusion for Control (A, B) and CH (C, D) hearts. *P<0.05 and **P<0.01 between high and low oxygen. N.S., non significantly different.

Figure 1 presents the energetic states of the perfused hearts of Control and CH mice under high and low oxygen conditions (respectively in [8] and the present work). Main observations deal with the difference in the effects of oxygen decrease on both energetic metabolites and contractile activity between control (figure 1A, 1B) and CH (figure 1C, 1D) hearts. As illustrated by the spectra presented in figure 1A, when Control hearts were perfused with low oxygen instead of high oxygen, a concomitant important increase in Pi and decrease in [PCr] (−23.4%, figure 1B) were measured, while ATP remained unchanged. Contractile activity was also affected with low oxygen as evidenced by the 46.2% decrease in RPP (figure 1B). This alteration in the contraction was the consequence of a simultaneous decrease in HR and in LVDP (figure 1B). By contrast with Control, CH heart bioenergetics was merely affected by the hypoxic insult. Indeed, in response to low oxygen, almost no changes in NMR spectrum of CH hearts were observed and only a slight increase in Pi was detected, while concentrations in PCr and ATP remained similar (figure 1C). Concerning contractile activity, while the decrease in HR under low oxygen conditions was comparable to Control, a significant increase in LVDP occurred (figure 1D). The consequence of these changes was a surprising constant RPP for CH hearts under low oxygen compared with high oxygen conditions (figure 1D).

**Figure 2:**
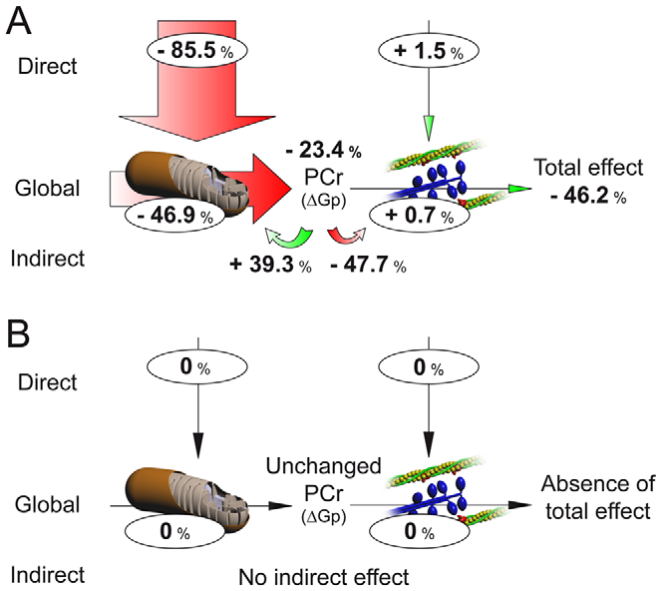
Modular Regulation Analysis of the effects of the decrease in oxygen availability on Control (A) and CH (B) mouse hearts. The size of the arrows is proportional to the effect of the decrease in oxygen availability, and the figures represent the effect expressed as % change from starting condition (high oxygen).

The application of Modular Regulation Analysis [22], [26] allows to quantify how decreasing oxygen availability triggers these changes in cardiac energetics in Control and CH hearts (see Text S1 for calculus). Results are presented in figure 2 both for Control (A) and CH (B) hearts. As described above, the total effect of low oxygen on Control hearts bioenergetics was a decrease of 46.2% in RPP, associated with a 23.4% decrease in [PCr] (figure 1B). This decrease by half of the contractile activity of the system is the consequence of two distinct effects induced by acute hypoxia on each module: (i) a direct effect of the decrease in oxygen on energy supply and demand, and (ii) an additional indirect effect on each module in response to the observed change in PCr concentration. Calculation of direct effect shows that decreasing oxygen availability strongly inhibits energy supply directly (−85.5% in our conditions) whereas energy demand is not affected (+1.5%) (figure 2A). These effects are responsible for the drop in PCr concentration (−23.4%), which in turn causes indirect effects on both modules, depending on their respective elasticities to changes in PCr. Thus, this drop in PCr induces a strong positive effect on energy-supply (+39.3%), that compensate in part for the strong direct negative effect induced by acute hypoxia on this module, and a strong negative effect on energy-demand (−47.7%) (figure 2A). The last information obtained from regulation analysis is the global response of heart contractile activity to the decrease in oxygen through each module. This global effect depends on the control coefficients and therefore quantifies how strongly the decrease in oxygen availability acts on the system through each module. Here, alteration induced by oxygen shortage on heart energetics essentially comes from energy supply inhibition with a global effect of −46.9% on contraction, whereas no effect is measured from energy demand (+0.7%) (figure 2A).

Regarding CH hearts, due to the absence of modification in RPP or [PCr] following oxygen decrease (figure 1D), modular regulation analysis reflected this absence of effect as illustrated in figure 2B. This absence of effect of acute hypoxia on CH hearts is the consequence of the insensitivity of energy supply to our conditions of hypoxia compared with Control (figure 2).

## Discussion

Constant heart muscle work requires efficient myocardial energetic regulation and matching between ATP consumption and production [27]. Cardiac metabolism principally rely on oxidative metabolism and adequate supply of oxygen and metabolic substrates is therefore a major prerequisite for normal cardiac bioenergetics [23]. Indeed, although heart can stand some degree of hypoxia, this situation is incompatible with its normal bioenergetic function, and ischemic heart disease with compromised oxygen supply to the myocardium is a common cause of heart failure [23], [28].

This idea is clearly illustrated in the present study by the concomitant severe decrease in contractile activity and PCr concentration measured in Control hearts perfused under low oxygen conditions compared with previously reported values [8] measured under high oxygen conditions (see figure 1). Similar alterations of cardiac energetics have already been described not only in isolated heart submitted to reduced O_2_-supply by decreasing coronary flow or PaO_2_ in the medium [29], [30], but also in the hypoxic myocardium of living rats [28].

However, in striking contrast with Control, CH hearts appeared insensitive to the decrease in oxygen and showed no modification either in PCr or in RPP when submitted to low oxygen perfusion. As evidenced by the regulation analysis of the effects of decreased oxygen availability on cardiac bioenergetics, this insensitivity of CH hearts to hypoxic stress is mainly the consequence of a surprising absence of direct effect of acute hypoxia on energy supply processes. This result indicates a specific modification in the bioenergetics of hypoxic hearts that compensate for decreased oxygen availability. Numerous studies previously showed that hearts adapted to chronic hypoxia are less prone to develop contractile dysfunctions, ventricular arrhythmia or myocardial infarction when submitted to acute oxygen deprivation (see [5], [12], [16]). Although the detailed mechanisms are poorly understood, adaptation of cardiac energy metabolism to hypoxic environment appears as a potential candidate in this higher resistance of CH hearts to hypoxic insult. Using the integrative tools of Modular Control Analysis (MoCA, [22]), we previously evidenced an increased kinetic response (elasticity) of energy production pathways to changes in [PCr] after chronic exposure to hypoxia [8], emphasizing a better responsiveness of energy supply to changes in ATP-demand in CH hearts. Advantages brought by this higher elasticity of energy-producing processes were not clearly figured out, but we proposed it as a mechanism developed to counteract the decrease in activity evidenced in CH hearts [8]. In the present study, when MoCA was applied to Control and CH hearts under conditions of reduced oxygen availability, a severe decrease in the elasticity of energy supply was measured in both groups compared with values measured under high oxygen conditions [8]. This result seems not surprising when considering the decrease in creatine kinase (CK) flux evidenced in hearts of living rats submitted to hypoxic ventilation [28], that could be responsible for an altered energy supply-demand matching. However, the higher elasticity of energy supply developed in CH hearts [8] appears to compensate this alteration and even under low oxygen conditions, energy supply elasticity remained twice higher as compared to Control. An important consequence is that, under low oxygen availability, the control exerted by energy-supply on contraction remains significantly lower in CH hearts compared with Control. This difference allows CH hearts to be characterized by a distribution of the control equally shared among energy supply and demand, as encountered in Control hearts under normal conditions of oxygenation [8].

**Figure 3:**
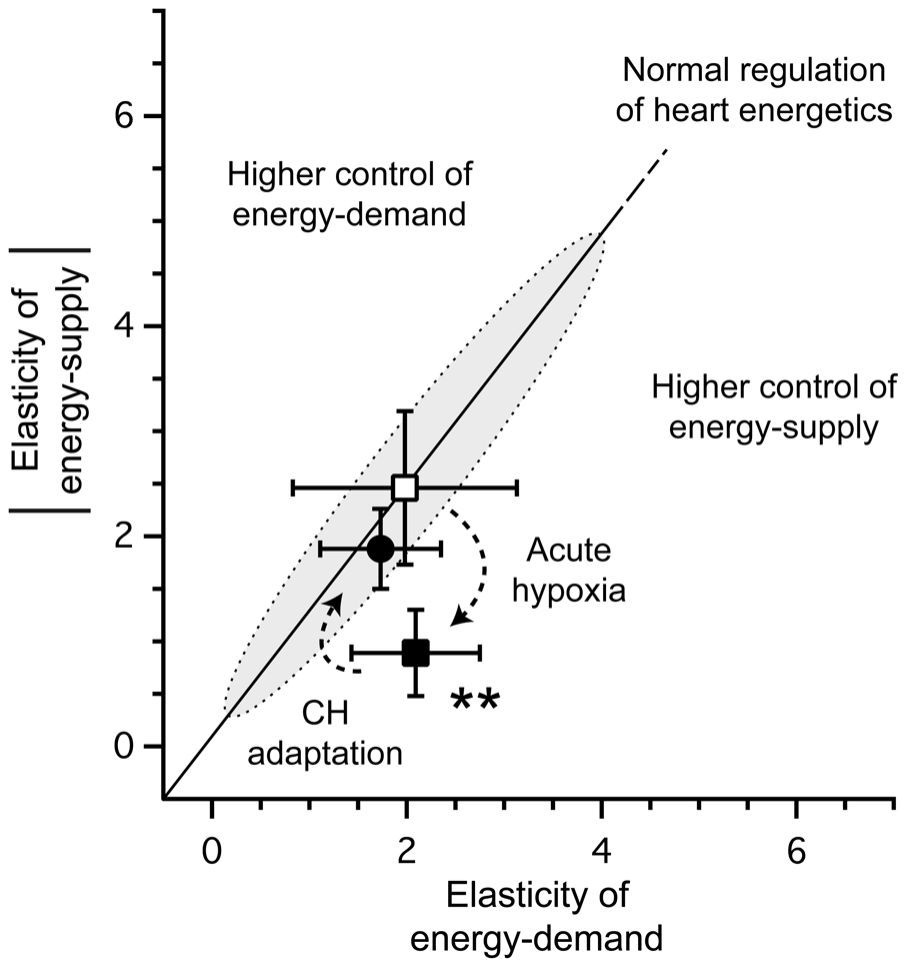
Elasticities plot of the adaptation of CH hearts to hypoxia. *Open square:* elasticities of Control hearts under high oxygen condition (energy supply: −2.46±0.73, energy demand: 1.98±1.15, values obtained from [8]). *Solid square:* elasticities of Control hearts in low oxygen condition (energy supply: −0.89±0.41, energy demand: 2.09±0.66). *Solid circles:* elasticities of CH hearts in low oxygen condition (energy supply: −1.88±0.38, energy demand: 1.73±0.62). *Shadowed zone* indicates normal control pattern (normal distribution of the control between energy supply and demand) in mouse heart energetics, as described in [8].

Values of elasticities and control coefficients of a metabolic system are subject to a number of constraints and inter-relationships [20]. This relation, illustrated in figure 3, provides the keys to understand how the elasticities of energy supply and demand affect the distribution of control coefficients on contraction. If we plot the value of the elasticity of a module against that of the other module, the line passing through this point and the origin describes the combinations of elasticities that give the same control pattern (figure 3). The effects of decreasing oxygen availability as well as CH adaptation on “normal” mouse heart energetics [8] could then be easily understood with this figure. Indeed, as illustrated, the decrease in energy supply elasticity induced by acute hypoxia on Control hearts, without any change in energy demand elasticity, move the system in a situation where control of energy supply on contraction becomes predominant (figure 3, acute hypoxia arrow). This situation is detrimental for cardiac bioenergetics and could be interpreted as energy deficiency, a situation encountered during heart failure [11], [31]. However, because of the higher elasticity of energy-supply developed in heart during chronic hypoxia, the effect of acute hypoxia is compensated (figure 3, chronic hypoxia arrow) and bioenergetics of CH heart keeps the same control distribution among energy supply and demand under low oxygen availability than healthy hearts under normal oxygen conditions. An important consequence is the absence of effect of decreased oxygen availability on energy-supply in CH hearts evidenced by Modular Regulation Analysis (figure 2).

Consequently, the marked increase in energy supply elasticity observed in CH hearts may be considered as an adaptive mechanism developed in the heart which counteracts the decrease in activity and protects CH hearts from oxygen shortage. Modifying the mechanisms responsible for energy production elasticity appears as a possible mean to maintain a balanced control pattern (“normal” control shared by energy-supply and demand) on contraction, and to ensure sufficient flexibility to adapt to new conditions. The most obvious process whose modifications may be responsible for the change in the kinetic interaction between energy production and PCr is the ANT-miCK-porin complex, which is at the interface between mitochondria and the energetic intermediates, including Cr/PCr. An improved coupling between all the components of this complex may lead to a more efficient matching of energy-supply to energy-demand. As suggested by this study, this improved communication between ATP supply and demand pathways is likely a key strategy to limit hypoxia-induced energetic alterations. This adaptation may ensure the preservation of the normal control pattern and regulation of energy supply and demand under the unfavorable conditions of oxygen availability.

Understanding the regulation and control of complex network of reactions requires analytical tools that take into account the interactions between individual network components controlling global network function. We believe that this holistic view of cardiovascular metabolism as a modular dynamic network may be conceptually useful for illuminating the basis of biological pathology and adaptation phenomena.

## Methods

### Ethics Statement

All animal work has been conducted according to relevant international and national guidelines in accordance with the recommendations of the Weatherall report, “The use of non-human primates in research”. All the protocols used were approved by our local ethics committee named Comité d'éthique régional d'Aquitaine. The corresponding license number is P.D. 3308010 (03/17/1999).

### Chronic Hypoxia

Female Swiss mice (6 weeks, 18–23 g) were separated into two groups. One group (chronic hypoxic mice, CH, n = 6) was exposed to a simulated altitude of 5500 m (barometric pressure 380 mmHg) in a hypobaric chamber for 21 days. The other group (normoxic mice, Control, n = 7) was maintained under ambient normoxic conditions (FiO_2_ = 21%), with the same 12∶12H light-dark cycles. Free access to a standard mice diet and water was allowed throughout the exposure period.

### Isolated Perfused Heart Perfusion

As previously described [8], once excised, hearts were immediately cannulated and retrogradely perfused at a constant pressure of 90 mmHg with warm (37°C), Krebs-Henseleit buffer (in mmol/L: NaCl 108, KCl 5.9, MgSO_4_ 1.2, NaHCO_3_ 25, EDTA 0.5, Mannitol 1.1, Glucose 10, Pyruvate 5 and CaCl_2_ 2.5, pH 7.35). The medium was equilibrated at a low PO_2_ (PO_2_ = 100–120 mmHg, medium air-equilibrated), to study hearts under conditions of reduced oxygen availability [32] compared to normal perfusion conditions (PO_2_ = 550–600 mmHg, medium saturated with 95% O_2_ - 5% CO_2_). The hearts were allowed to beat spontaneously. Heart rate (HR) and left ventricle developed pressure (LVDP) were measured continuously with a fluid-filled balloon inserted into the left ventricle. Mechanical performance of the heart was evaluated as the product of HR and LVDP (RPP, in mmHg/min). All measurements were performed after a 20 min stabilization period.

### NMR Measurement

Pulsed Fourrier-transformed ^31^P NMR spectra were obtained using a 11.7 T superconducting magnet (Bruker, Karlsruhe, Germany). Hearts were inserted into a heated (37°C) ^1^H/^31^P double-tuned 10 mm probe. The probe was tuned to the 202.4 MHz phosphorus resonance frequency. Partially saturated ^31^P NMR spectra (13 µs RF pulse, 60° flip angle, 2 s repetition time, 4096 data points, reception bandwidth 6460 Hz, 150 acquisitions) were obtained in 5 min without proton decoupling. The resonance area corresponding to ATP, PCr, and inorganique phosphate (Pi) were fitted to Lorentzian functions and calculated using a commercially available program (Igor Pro, Wavemetrics,). By comparing the areas under the peaks from fully relaxed (recycle time 17.5 s) and those of partially saturated (recycle time 2 s) spectra, correction factors for saturation were calculated for ATP (1.0), PCr (1.2) and Pi (1.15). The β-ATP resonance area was fixed at 11.8 mmol/L [33] for the first spectra and and used for internal calibration of the NMR spectra.

### MoCA (Modular Control and Regulation Analysis)

**Figure 4:**
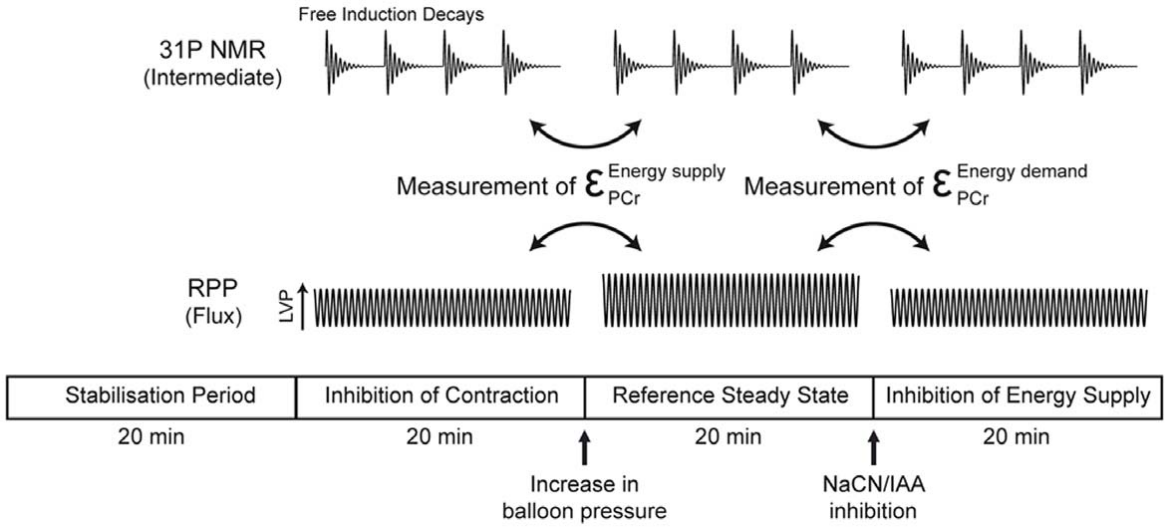
Experimental protocol for the measurement of both energy supply and demand elasticities in each heart. All measurements were performed after a 20 min stabilization period.

The principles of the application of MoCA on muscle bioenergetics have been extensively described elsewhere for heart [8], [22], [34], [35] and skeletal muscle [36], [37] and only a short description will be found here (for a thorough description of the MoCA approach and the equations used, see [22] and Text S1). MoCA describes heart energetics on the basis of a supply-demand system (energy-production and -consumption pathways connected by energetic intermediates). Assessment of specific changes in [PCr] (representative of the changes in ΔGp) by ^31^P-NMR spectroscopy and in contractile activity allows the quantification of the integrated kinetic response (the elasticity) of supply and demand modules. In the present study, elasticities of energy supply and demand of Control and CH hearts under low oxygen perfusion were determined similarly to [8] respectively after increase in balloon volume and injection of low cyanide concentration (NaCN, 0.3 mM) combined with iodoacetic acid (IAA, 75 µM). The experimental protocol allowing the measurement of both elasticities for each heart is summarized in Figure 4. The control coefficients of both modules on heart contraction were calculated from these elasticities according to the summation and connectivity theorems [20].

Moreover, MoCA may potentially provide the full description and quantification of the integrated regulatory effects of any modulation of heart contractile activity (Regulation Analysis, [22], [26]). Here we applied this analysis to study the effects of the lowering in oxygen on heart energetics of Control and CH mice. Effects of the lowering in oxygen were quantified in terms of Direct Effect (total effect on the energetic system minus the Indirect Effect on each modules due to PCr changes) and Global Effect [22].

### Enzyme Assays

At the end of each experiment, hearts were rapidly freeze-clamped with tongs cooled in liquid nitrogen and stored frozen at −80°C for later determination of enzyme activity. Immediately after thawing, tissue samples were minced with scissors, placed into ice-cold solution (50 mg wet weight per 1 mL) containing (mM): Tris-HCl buffer, 0.1; EGTA, 1; pH 8.1, and homogenized with an Ultra-Turrax homogenizer. Tissue homogenates were incubated for 60 min at 0°C for complete enzyme extraction and centrifuged at 13,000×g for 20 min. The supernatant was used for enzyme determination. Citrate synthase (E.C. 4.1.3.7) was determined by the method of Srere [38]. Assays were performed at 30°C. Each determination was carried out in triplicate.

### Statistical Analysis

Experimental values are reported as means ±SD. Statistical comparison between groups (CH vs. Control) and perfusion conditions (high vs. low oxygen) was performed by one-way ANOVA and post-hoc Tukey HSD tests. p<0.05 and p<0.01 stand for significant difference levels.

## Supporting Information

Text S1 Application of Modular Control and Regulation Analysis to isolated perfused heart of mouse.
(0.05 MB DOC)
