# Supplementary material for: Improved Energy Supply Regulation in Chronic Hypoxic Mouse Counteracts Hypoxia-Induced Altered Cardiac Energetics
Source: PLoS One. 2010 Feb 18;5(2):e9306. doi: 10.1371/journal.pone.0009306 (PMC2823784; doi:10.1371/journal.pone.0009306)
Supplement: Text S1 — Application of Modular Control and Regulation Analysis to isolated perfused heart of mouse. (0.05 MB DOC) [file pone.0009306.s001.doc]

**TEXT S1: APPLICATION OF MODULAR AND REGULATION ANALYSIS TO ISOLATED PERFUSED HEART OF MOUSE**

**Modular Control Analysis (MoCA)**

The principles of the application of MoCA on muscle bioenergetics have been extensively described elsewhere for heart [1-5] and skeletal muscle [6,7]. MoCA approach considers heart energetics as two modules representing energy supply (all the steps from substrates and oxygen supply to mitochondrial phosphocreatine production through PCr-CK system) and energy demand (mainly myosin ATPases and reticulum sarcoplasmic ATPases) pathways, linked by the energetic intermediates. Changes in [PCr] are representative of the changes in the energetic intermediates (and of the resulting free energy of ATP hydrolysis (ΔGp)) [2,3]. Kinetic interaction (elasticity, ε) of each module toward [PCr] was calculated as the relative change in contractile activity (rate pressure product, RPP) divided by the relative change in [PCr] in response to slight modulation of the other module [2] :

In the present study, elasticity of energy supply and demand of Control and CH hearts under low oxygen perfusion were determined similarly to [1] respectively after balloon increase (Franck Starling effect) and injection of low cyanide concentration (0.3 mM NaCN) combined with iodoacetic acid (IAA, 75 μM). For the Frank Starling effect, an initial LV volume (typically 10 µL and 7.5 µL for control and CH hearts respectively) was set by adjusting the balloon volume to yield a LV end-diastolic pressure of ~6-8 mmHg. Then, to obtain a comparable modulation of the energy-demand module, the increase in balloon pressure was performed taking into account the different LV volumes of the two groups previously measured by MRI [8] (i.e. a 20-25% smallest LV volume for CH hearts): in consequence, increase in balloon pressure was respectively + 10 µL and + 7.5 µL for Control and CH hearts. The experimental protocol allowing the measure of both elasticities for each heart is summarized in the Figure 4 of the original paper. The control coefficients of both modules on heart contractile activity () were then calculated from the elasticities according to the summation and connectivity theorems [9,10]:

**Modular Regulation Analysis**

Modular regulation analysis [2,11-13] provides a quantitative description of the effect of any effectors (drug, pathology, etc) on each internal module of a system, resulting in a global modification of the intermediate concentration and energetic flux of the system (e.g. [PCr] and RPP for cardiac energetics). Here we applied this analysis to study the effects of the lowering in oxygen on heart energetics of Control and CH hearts. The responses of the perfused heart to low oxygen (effect of low oxygen on metabolite (PCr) and energy flux (RPP)) were analyzed using the principles of Top Down Regulation Analysis established in [11] and applied by us on perfused rat heart [2]. In the present study, because of the change in elasticity induced by low oxygen condition, a mean elasticity was taken to perform the analysis. Effects of the lowering in oxygen were quantified in terms of Direct Effect (IE, quantify the direct effect on each module, defined as the total effect on the energetic system minus the Indirect Effect on each modules due to PCr changes) and Global Effect through the modules (quantify changes of the energetic flux due to direct effects) [2].

Indirect Effect on Module =

Direct Effect on Module (IE) =

Global Effect through Module =

References

1. Calmettes G, Deschodt-Arsac V, Thiaudiere E, Muller B, Diolez P (2008) Modular control analysis of effects of chronic hypoxia on mouse heart. Am J Physiol Regul Integr Comp Physiol 295: R1891-1897.

2. Diolez P, Deschodt-Arsac V, Raffard G, Simon C, Santos PD, et al. (2007) Modular regulation analysis of heart contraction: application to in situ demonstration of a direct mitochondrial activation by calcium in beating heart. Am J Physiol Regul Integr Comp Physiol 293: R13-19.

3. Diolez P, Raffard G, Simon C, Leducq N, Dos SP, et al. (2002) Mitochondria do not control heart bioenergetics. Mol Biol Rep 29: 193-196.

4. Korzeniewski B, Deschodt-Arsac V, Calmettes G, Franconi JM, Diolez P (2008) Physiological heart activation by adrenaline involves parallel activation of ATP usage and supply. Biochem J 413: 343-347.

5. Vogt AM, Poolman M, Ackermann C, Yildiz M, Schoels W, et al. (2002) Regulation of glycolytic flux in ischemic preconditioning. A study employing metabolic control analysis. J Biol Chem 277: 24411-24419.

6. Arsac LM, Beuste C, Miraux S, Deschodt-Arsac V, Thiaudiere E, et al. (2008) In vivo modular control analysis of energy metabolism in contracting skeletal muscle. Biochem J 414: 391-397.

7. Beuste C, Miraux S, Deschodt-Arsac VJ, Thiaudiere E, Franconi JM, et al. (2009) Modular regulation analysis of integrative effects of hypoxia on the energetics of contracting skeletal muscle in vivo. Biochem J 420: 67-72.

8. Miraux S, Calmettes G, Massot P, Lefrancois W, Parzy E, et al. (2009) 4D retrospective black blood trueFISP imaging of mouse heart. Magn Reson Med 62: 1099-1105.

9. Kacser H, Burns JA (1973) The control of flux. Symp Soc Exp Biol 27: 65-104.

10. Fell DA (1992) Metabolic control analysis: a survey of its theoretical and experimental development. Biochem J 286 ( Pt 2): 313-330.

11. Ainscow EK, Brand MD (1999) Quantifying elasticity analysis: how external effectors cause changes to metabolic systems. Biosystems 49: 151-159.

12. Brand MD (1997) Regulation analysis of energy metabolism. J Exp Biol 200: 193-202.

13. Brand MD (1996) Top down metabolic control analysis. J Theor Biol 182: 351-360.
